# Medical work Assessment in German hospitals: a Real-time Observation study (MAGRO) – the study protocol

**DOI:** 10.1186/1745-6673-4-12

**Published:** 2009-06-08

**Authors:** Stefanie Mache, David A Groneberg

**Affiliations:** 1 Institute of Occupational Medicine, Charité – School of Medicine, Free University and Humboldt University, Thielallee 69-73, 14195 Berlin, Germany; 2 Department of Medicine/Psychosomatics, Charité – School of Medicine, Free University and Humboldt University, Luisenstrasse 13a, 10117 Berlin, Germany

## Abstract

**Background:**

The increasing economic pressure characterizes the current situation in health care and the need to justify medical decisions and organizational processes due to limited financial resources is omnipresent. Physicians tend to interpret this development as a decimation of their own medical influence. This becomes even more obvious after a change in hospital ownership i.e. from a public to a private profit oriented organization. In this case each work procedure is revised.

To date, most research studies have focused mainly on differences between hospitals of different ownership regarding financial outcomes and quality of care, leaving important organizational issues unexplored. Little attention has been devoted to the effects of hospital ownership on physicians' working routines.

The aim of this observational real time study is to deliver exact data about physicians' work at hospitals of different ownership.

**Methods:**

The consequences of different management types on the organizational structures of the physicians' work situation and on job satisfaction in the ward situation are monitored by objective real time studies and multi-level psycho diagnostic measurements.

**Discussion:**

This study is unique in its focus. To date no results have been found for computer-based real time studies on work activity in the clinical field in order to objectively evaluate a physician's work-related stress. After a complete documentation of the physicians' work processes the daily work flow can be estimated and systematically optimized. This can stimulate an overall improvement of health care services in Germany.

## Background

### The current situation for ward physicians working in German hospitals

The German health care system is undergoing substantial changes in terms of general medical supply in hospitals and outpatient clinics and the role of public health insurance. Currently hospitals are dealing with declining financial support. Because of this there is a higher doctor-patient ratio, recently introduced work time schedules and a decrease in personnel expenditure.

After the implementation of structured and more formalized treatment procedures, quality of care and economic aspects should improve. But for physicians it is becoming increasingly difficult to treat patients adequately. The aforementioned framework has a tremendous influence on every day medical services. Individual medical treatment of patients is becoming increasingly unusual under the current circumstances. How much time is spent on non-medical issues such as administrative work is not clear because only subjective data is available.

Many doctors complain about their current work situation [1]. The most frequent complaints include: working overtime, doing extended shift work and on-call duties at short notice, missing internal coordination and communication of working routines and increasing administrative and documentations tasks are named most frequently [2].

Further reasons for physician dissatisfaction are enormous workloads leading to massive time pressure and decreased treatment periods [3-5]. More work strain is to be expected due to staff cuts and thus an increase in work intensity. Work obstacles such as incomplete patient documentation and constant interruptions lead to a deterioration of medical tasks [3,4].

Occupational safety problems were primarily found in the field of accident risk or exposure to noise or chemical contamination. This situation has now changed and physicians are more at risk of suffering from psychological stress than other factors [3,4].

A further problem concerns work time organization. According to a study of the German Institute for Economics (2003), young physicians work 55.3 hours on average per week. Only 40% of German hospitals introduced new work time models [6] after the judgment of the European Union from 9 September 2003 which refers to the problematic situation of the medical profession in German hospitals. Long working hours not only have an effect on physicians' work and their well-being but also on the quality of patient care.

Research results clarify that it is becoming increasingly important to analyze the workflow in hospitals objectively and precisely. Nevertheless, at this time, heavy workloads are generally accepted and objective task analyses are not being carried out. So far analyses on this topic were based on questionnaires and statements of physicians [4]. However it can be assumed that there is a possible discrepancy between the results of objective and subjective observation [7].

### Consequences of hospital privatization on the occupational situation of physicians

Until recently hospitals were predominantly publicly owned. This has changed dramatically in the recent past. There is a tendency toward privatization in our society and hospitals are no exception [8].

Privatization may have, but does not necessarily have to have an influence on workload. The actual effect on work conditions is hard to estimate and is, therefore, the aim of this study. In a report published by The German Physicians' Union results were not consistent [9].

Currently no scientific results either related to the professional situation after privatization or general changes in a physician's work role have been found. This study plans to close the gap.

The German Medical Association claims to have carried out a more qualitative analysis of privatized hospitals in 2007 [9]. Comparisons beyond economic data are greatly needed. Variables that should be researched are the quality of workflow, patient satisfaction, amount of sick leave, and the fluctuation of employees.

### Job task analysis method

To evaluate work flow quantitatively an empirical measurement is needed. Usually a job task analysis is utilized. The general purpose of job task analysis is to document the requirements of a job and the work performed. Task analysis is performed as a basis for later improvements including: definition of a job domain, developing performance appraisals, selection systems, training needs assessment, and compensation plans [10].

The method is defined by an exact observation of every single activity within a given period. Originally a stopwatch, paper and pencil were utilized. To ensure a smooth procedure and a better comparability as well as a subsequent legibility of observed tasks, symbolization was introduced to represent certain events and to create a system of categories. These patterns are the most important qualitative basis for this analysis.

Some of these techniques are more focused on recording body positions and movements, such as the WinOWAS [11], TRAC (Task Recording and Analysis on Computer [12] or PEO (Portable Ergonomic Observation Method [13]. The current procedure is to connect the protocol to electronic data software programs. This allows repeated inspection and further data analysis. The calculation of statistical parameters and graphic models are easily carried out.

Further improvements of such methods will be included in this study:

• Definition of a system of categories for the physician's task analysis

• Creation of a user-friendly interface

• Improvement in the mode of interaction during the recording procedure

## Aims and objectives

### Research and health-political significance

The overall aim of this real-time study is to evaluate exact data relating to work.

A short term goal of the investigation is to provide referential values of different medical specialties in order to define accurate job task profiles of different physician groups. In order to achieve an effective comparison of the groups, physicians with similar specialties should be included, but working in hospitals of different ownership (e.g., private vs. public).

In the context of the investigation a multimodal diagnostic measurement is to be used to evaluate physicians' work load and demands. In order to reduce psychological and physical demands effectively, it is necessary to identify ranges of stress.

On one hand, objective workloads must be determined independent of the physicians' subjective perception. On the other hand, personal points of view must be brought into focus by asking how specific job characteristics influence a physician's well being and job satisfaction as well as what kind of social and organizational resources might have a positive effect on managing work load.

In the context of the monitoring the following main questions need to be answered.

1. Do significant differences exist regarding the organization of work flow between physicians of the same medical discipline, but from hospitals of different ownership?

2. Do different work and organization models have effects on the occupational situation (job demands, state of health) and work satisfaction of physicians?

3. What kinds of workload factors can be observed when comparing different work and organization models?

4. Do specific factors exist that have a substantial influence on a physician's job satisfaction?

5. Do occupational resources have an impact on the ability to work?

6. Do work obstacles which have an influence on physicians' work situation and job satisfaction exist?

### Development of a work assessment method to evaluate physicians' workflow

Exclusive observations and questioning would increase the danger of incorrectly evaluating work conditions and of insufficiently addressing the actual work situation. With the help of a computer-based recording methodology, a possibility is given to counteract this bias. Therefore detailed computer-based task observations should be implemented in the work analysis.

Keeping that in mind, the second aim of this study is to examine and to develop computer software. Thus a progressive work analysis procedure could be available for further research. This study will focus on the following:

• The procedure which considers different areas of a physicians' work situation in hospital departments (work flow, organizational aspects, etc.).

• The procedure which can be implemented in different medical specialties (e.g., Internal medicine, Neurology, Pediatrics, etc.) and hospital types (public, private hospitals, etc.).

## Methods

### Sample and Recruitment

**Figure 1:**
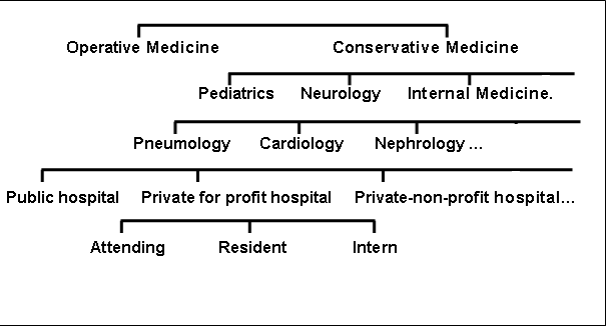
**Study Design**.

Physicians of different medical specialties will be included in the study (i.e., Neurology, Psychiatrics, Surgery, Pediatrics, Internal medicine). In addition, they will be recruited from hospitals of different ownership types (private for-profit, private non-profit, public) (Fig. 1).

After the research group will have had contacted hospital supervisors, a selection of physicians will take place on a voluntary basis. The comparative samples are composed of physicians of the same medical specialty; however, they work at hospitals of different ownership. A satisfactory comparability of the groups will thus be achieved.

### Instruments

#### Objective task-analysis

Objective task analysis is based on data of physicians' work in hospital departments.

The data of the study will represent the basis for load analyses. Only via objective task analyses can a differentiation be made among the participating physicians with regard to their affiliation with very stressful or less stressful jobs.

**Figure 2:**
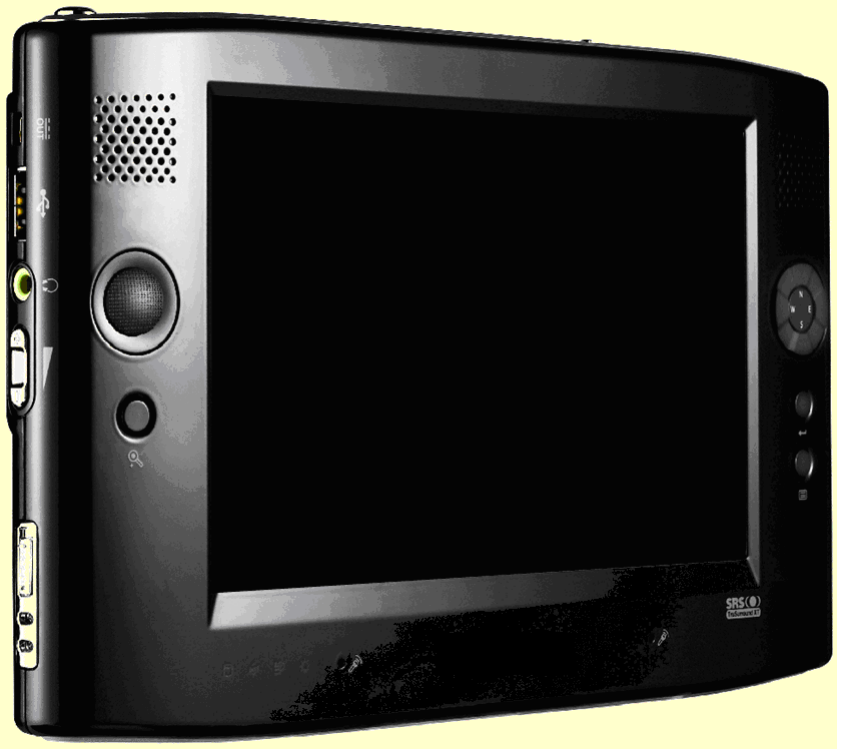
**Ultra Mobile Computer**.

With the help of the developed activity acquisition program workflow can be registered objectively by mobile computer-based collection equipment (Ultra Mobile PC) (Fig. 2).

The analytical apparatus can be carried in one hand and possesses a touch screen. By touching the surface of the mobile computer an exact time is registered.

When the assessment is complete, data will be transferred to another PC and evaluated statistically and graphically: the number of individual occurrences of each task, mean duration of each occurrence, the total time (in seconds) spent on each task, task category, and the time expenditure for all tasks will be counted.

To collect meaningful information about physicians' workflow at least 20–30 physicians per medical specialty should be observed during an entire work week. Individual weekdays should be represented equally, so that a comparison of the medical activity is possible for all weekdays.

Afterwards the collected data can be interpreted as referential values for the physicians' work. Early and late shifts are equally represented.

During the task analysis the following aspects should be registered:

• The kind of work activity

• Duration and frequency of the tasks

• Work environment (lighting, room temperature, noise level)

• Interruptions (frequency, duration, type)

• Rest periods (time, duration, location)

#### A questionnaire study on perceived job demands and work stress

In addition to the above a comparison analysis is to be carried out regarding objective and subjective perception of the work situation. Since workload can only be determined and judged by the coworkers themselves, physicians will be asked to fill out a questionnaire, which includes questions on each individual's perception of their work conditions and workload.

The German version of the Copenhagen Psychosocial Questionnaire (COPSOQ) will be used to assess job-related factors at work [14]. Various aspects of work, for example, job demands (e.g., quantitative demands); job resources (i.e., quality of leadership, social support) as well as job outcomes (i.e., state of health) are captured by the COPSOQ. The version used in this survey comprised 16 scales [14].

The questionnaire will be presented as a pencil-and-paper version. Physicians will be requested to post it in a box placed in their departments within three weeks.

### Statistical data analysis

The following statistical analysis will be carried out dependent on the hypotheses:

descriptive statistics and ANOVAs will be calculated to examine whether there are differences in work conditions as well as in job satisfaction between the three ownership types.

Hierarchical regression analysis will be used to analyze the degree to which job satisfaction can be explained by physicians' job demands and resources. To examine the differences in the effects of job demands and resources between the hospitals, separate analyses will be performed for each type of hospital ownership.

All p-values given will be two-tailed. A p-value of less than .05 will be considered significant. Values will be given as mean and standard deviation (SD). Data will be calculated using the SPSS^® ^software package for social sciences; Version 17.0.

## Discussion

At this time no results have been found for computer-based real time studies on work activity in the clinical field in order to objectively evaluate a physician's work-related stress.

It is a central and a long-term goal to develop possibilities for improvements to optimize physicians' work flow. Reorganization of work flow should lead, for example, to a respite from non-medical tasks. Periods of high workload, which lead to an additional burden, can thus be avoided. As a result the physician's job satisfaction will be increased and better patient care can be ensured. All things considered, this study can stimulate an overall improvement of health care services in Germany.

## Competing interests

The authors declare that they have no competing interests.

## Authors' contributions

SM and DAG conceived and designed the study. SM wrote the manuscript. SM and DAG contributed substantially to its revision.
